# Effectiveness of managed gene flow in reducing genetic divergence associated with captive breeding

**DOI:** 10.1111/eva.12331

**Published:** 2015-11-04

**Authors:** Charles D Waters, Jeffrey J Hard, Marine S O Brieuc, David E Fast, Kenneth I Warheit, Robin S Waples, Curtis M Knudsen, William J Bosch, Kerry A Naish

**Affiliations:** 1 School of Aquatic and Fishery Sciences, University of WashingtonSeattle, WA, USA; 2 Northwest Fisheries Science Center, National Marine Fisheries Service, National Oceanic and Atmospheric AdministrationSeattle, WA, USA; 3 Yakama Nation FisheriesToppenish, WA, USA; 4 Washington Department of Fish and WildlifeOlympia, WA, USA; 5 Oncorh ConsultingOlympia, WA, USA

**Keywords:** captive breeding, domestication selection, genome-wide survey, managed gene flow, supportive breeding

## Abstract

Captive breeding has the potential to rebuild depressed populations. However, associated genetic changes may decrease restoration success and negatively affect the adaptive potential of the entire population. Thus, approaches that minimize genetic risks should be tested in a comparative framework over multiple generations. Genetic diversity in two captive-reared lines of a species of conservation interest, Chinook salmon (*Oncorhynchus tshawytscha*), was surveyed across three generations using genome-wide approaches. Genetic divergence from the source population was minimal in an integrated line, which implemented managed gene flow by using only naturally-born adults as captive broodstock, but significant in a segregated line, which bred only captive-origin individuals. Estimates of effective number of breeders revealed that the rapid divergence observed in the latter was largely attributable to genetic drift. Three independent tests for signatures of adaptive divergence also identified temporal change within the segregated line, possibly indicating domestication selection. The results empirically demonstrate that using managed gene flow for propagating a captive-reared population reduces genetic divergence over the short term compared to one that relies solely on captive-origin parents. These findings complement existing studies of captive breeding, which typically focus on a single management strategy and examine the fitness of one or two generations.

## Introduction

The genetic risks associated with captive breeding are widely recognized. Rearing conditions may result in the relaxation of natural selective pressures and impose artificial selection favoring individuals suited for captivity instead of the natural environment (Frankham 2008). Additionally, genetic drift and inbreeding may reduce genetic diversity within captive populations (Fraser 2008; Naish et al. 2008). Thus, captive and wild populations may become genetically divergent over time, and individuals raised in captivity for supplementation purposes may be less effective in contributing to the population’s long-term viability than their wild-born counterparts (Fraser 2008; Jule et al. 2008; Laikre et al. 2010). Yet, captive breeding may be the only feasible tool for species recovery and conservation, particularly as rates of extinction increase (Pimm et al. 2006; Burkhead 2012). Therefore, there is a pressing need to develop and test approaches that minimize these risks over sufficient generations for population recovery to occur (Williams and Hoffman 2009). Theoretical treatments (Lynch and O’Hely 2001; Duchesne and Bernatchez 2002; Ford 2002) suggest that intentional promotion of gene flow from wild to captive populations may mitigate genetic divergence caused by genetic drift, inbreeding, and selection during captive breeding (Mobrand et al. 2005; Frankham 2008; Paquet et al. 2011). This strategy, which we refer to here as managed gene flow, should be investigated in empirical settings to determine whether the approach reduces genetic changes that could adversely affect fitness and limit restoration success.

Supportive breeding is a form of captive breeding intended to enhance the sizes of populations in their natural environment (Ryman and Laikre 1991). In such programs, a fraction of a population is taken into captivity for reproduction, and their progeny are released back into the natural environment to join wild-born conspecifics. This method avoids captive rearing for the full life cycle and might aid *in situ* population recovery if captive offspring have high reintroduction success. Supportive breeding using hatcheries has become an important component of many recovery plans for anadromous Pacific salmon on the West Coast of North America, where wild populations have steadily declined due to anthropogenic disturbances (National Research Council 1996). Historically, many hatcheries used returning hatchery fish for broodstock, but potential divergence of these fish from the wild population might result in decreased fitness following interbreeding in the natural environment (Busack and Currens 1995; Campton 1995). Recent management reforms aimed at mitigating negative effects of captive rearing have led to the widespread use of locally-derived broodstock and implementation of managed gene flow between the hatchery and naturally-derived components (Mobrand et al. 2005; Paquet et al. 2011).

There are several concerted research efforts aimed at understanding the impacts of supportive breeding on wild populations and the effectiveness of recent hatchery reforms. Pedigree-based studies have provided evidence of reduced reproductive success of hatchery-origin fish spawning in the natural environment relative to that of wild fish in the same cohort (Araki et al. 2007; Milot et al. 2013; Christie et al. 2014). However, the significance of reduced fitness of first-generation hatchery fish to the future viability of supported populations is unclear. The captive environment typically facilitates the reproduction of most individuals regardless of their fitness in the natural environment (Neff et al. 2011), but subsequent selection on *F*_1_ offspring released in the wild might remove maladaptive individuals (Baskett and Waples 2013) and reduce their effects on population fitness. Alternatively, ongoing broodstock collection and reduced fitness of *F*_1_ hatchery offspring might have long-term negative demographic and genetic effects on the wild component (Ryman and Laikre 1991; Ford 2002; Baskett and Waples 2013). It is also challenging to measure the relative fitness of the offspring of hatchery fish that spawned in the wild (the *F*_2_ generation, born in the wild) because matings that produce no offspring are typically unobserved (Araki et al. 2009), and subsequent analyses can have reduced power to detect an effect (Christie et al. 2014). Finally, many pedigree studies are necessarily opportunistic because they often rely on established systems, and specific outcomes might depend on local management objectives. It is therefore important to develop complementary experimental methods that examine the consequences of captive rearing on genetic diversity in hatchery-produced fishes, and investigate the potential for managed gene flow to reduce their genetic divergence from wild fish.

A powerful way to evaluate the use of managed gene flow in supportive breeding would be to study a system that is comparative in nature, namely one that maintains a captive line that has been deliberately kept separate from wild individuals, and a second line where wild individuals are used exclusively as captive broodstock. Variation in each generation of the captive lines could be compared to the original founding population using genome-wide surveys, which offer a means to broadly estimate rates of genetic change. Such a comparison would provide insight into the range of possible outcomes of managed gene flow and refine understanding on the optimal longevity of supportive breeding programs.

**Figure 1:**
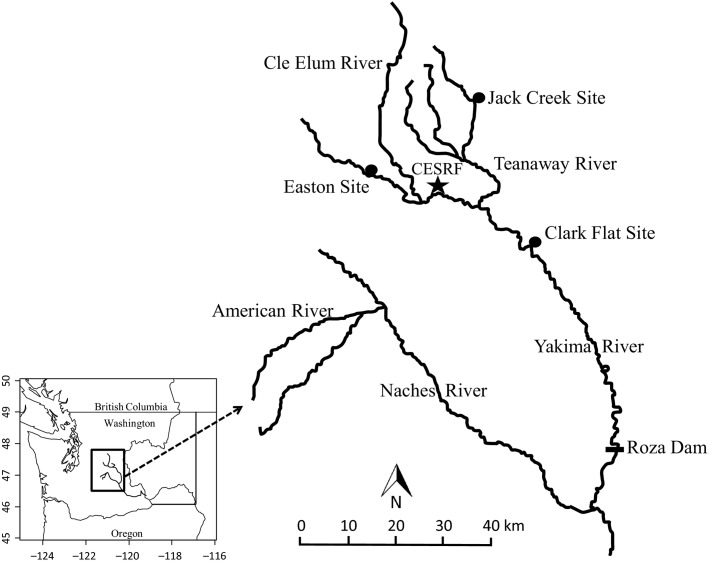
Map of the Yakima River system. The three spring Chinook salmon populations spawn in the American River, the Naches River, and the upper Yakima River above Roza Dam. The upper Yakima population is the target of the Cle Elum Supplementation and Research Facility (CESRF). All adults returning to the upper Yakima River are sampled at Roza Dam and allowed to spawn naturally (natural-origin and integrated line fish) or are removed from the system (all segregated line fish). Spawning and rearing for the hatchery lines occurs at CESRF. Prior to outmigration in spring, juveniles are transferred to the Easton, Jack Creek, and Clark Flat acclimation sites, where they are held for approximately two months before volitional release.

**Figure 2:**
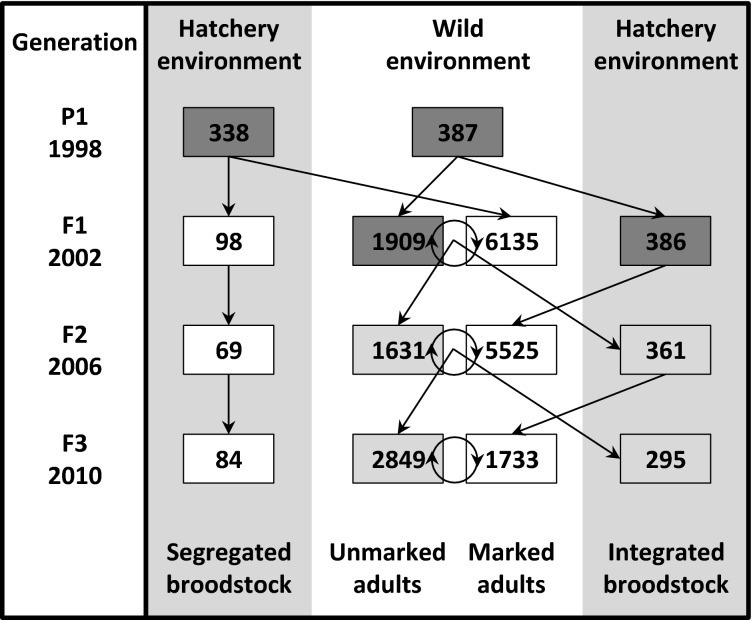
Schematic illustrating the initiation (the founding *P*_1_ generation) and subsequent broodstock management (*F*_1_–*F*_3_ generations) for the integrated and segregated hatchery lines of anadromous Chinook salmon that were surveyed. Numbers given in each box are the numbers of spawners (wild environment) and the number of broodstock (hatchery environment) for each brood year surveyed. Linear arrows denote the contribution of wild spawners or hatchery broodstock to the subsequent generation. Circular arrows represent unobserved mating between wild-born (unmarked) and hatchery-born (marked) spawners in the wild environment. Dark gray boxes represent wild adults, light gray boxes represent natural-origin adults with hatchery, wild, or hybrid ancestry, and white boxes represent adults born in the hatchery. Only brood years sampled are illustrated, but the same design is implemented each year. Chinook salmon are semelparous but have overlapping generations – approximately 80–90% of adults at CSERF are four-year-olds.

A hatchery program at the Cle Elum Supplementation and Research Facility (CESRF) was initiated in 1997 in response to declining anadromous spring Chinook salmon returning to the Yakima River, a tributary of the Columbia River, USA (Fig. 1). Wild adults were collected for founding broodstock from the upper Yakima River population from 1997 to 2002. Beginning with brood year 2002, the hatchery population was divided into a segregated (SEG) line, which was not allowed to interbreed with the source population, and an integrated (INT) line, which was allowed to spawn in the river (Fig. 2). All first-generation hatchery fish from the integrated line were allowed to spawn naturally. The proportion of hatchery fish from the integrated line spawning in the natural environment has varied between 0.2 and 0.76 (mean = 0.56) from 2001 to 2013 (Fast et al. 2015). There is indirect evidence that these hatchery fish successfully contributed to the natural population: hatchery- and natural-origin fish had similar distributions on the spawning grounds (Dittman et al. 2010), and redd (nest) abundance and spatial distribution has increased (Fast et al. 2015). Fish from the integrated and segregated hatchery lines are raised in the same facility but are differentially marked for external identification. The two lines have been reared for three generations, and DNA samples have been collected from every adult fish used as broodstock since the inception of the program (fish are intercepted at Roza Dam, Fig. 1). Thus, this system provides an extraordinary opportunity to experimentally evaluate efforts to reduce genetic change in captive management.

The aim of our study was to determine whether managed gene flow between natural and captive environments was effective at reducing genetic divergence relative to a wild founding population over three generations in a Chinook salmon supportive breeding program. We used a population genomic approach to survey genetic variation at 9410 polymorphic restriction site-associated DNA (RAD) markers, including 4405 markers that were anchored to a dense linkage map representing all 34 chromosomes. These markers were first used to test whether genetic divergence occurred in the integrated and segregated hatchery lines over each generation since founding, and to determine whether managed gene flow was effective at reducing differentiation. We also investigated potential mechanisms underlying observed changes. Changes due to genetic drift were examined using estimates of effective number of breeders. Molecular signatures of adaptive divergence, possibly indicative of domestication selection, were detected using multiple outlier analyses. By combining these results, we empirically validated an approach aimed at minimizing potential deleterious genetic effects of supportive breeding, and identified possible causes that might lead to reduced fitness of captive-reared individuals.

## Materials and methods

### Study system

The Yakima River is a tributary of the Columbia River located in south-central and eastern Washington State, USA (Fig. 1). The basin is home to three genetically distinct populations of spring, stream-type Chinook salmon (*Oncorhynchus tshawytscha*) in the upper Yakima River, the Naches River, and the American River (Fig. 1; Busack and Marshall 1991). Adult Chinook salmon return to the basin in spring, spawn in fall, and their offspring spend an entire year in freshwater before migrating to the ocean.

**Figure 3:**
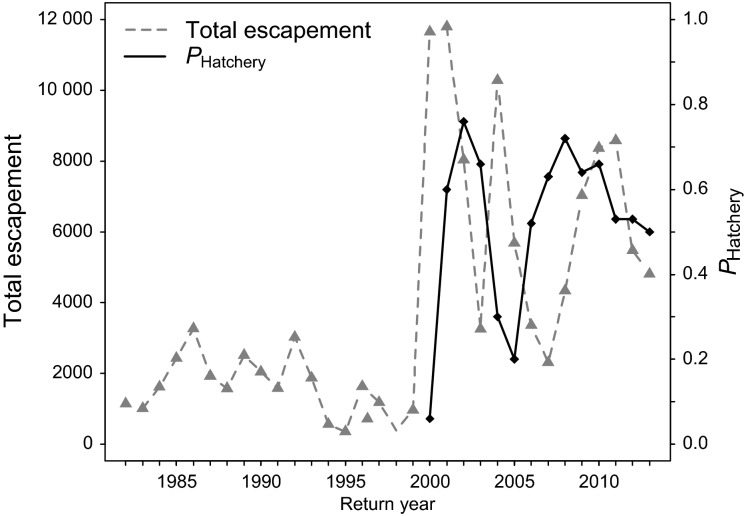
Total annual escapement of adult Chinook salmon in the upper Yakima river (gray, dashed line) and the proportion of spawners that are of hatchery origin (black line).

Annual returns of anadromous wild spring Chinook salmon to the Yakima River averaged approximately 1600 individuals in the 1980s and 1990s (Fig. 3). These population sizes represent a decline of 98% from estimates of historical returns (ref. in Lichatowich and Mobrand 1995). The hatchery program at CESRF was explicitly designed to test whether supportive breeding could increase harvest and production in the upper Yakima River Chinook population while minimizing ecological and genetic risks associated with captive rearing (RASP (Regional Assessment of Supplementation Planning) 1992).

Returning wild adults from the upper Yakima population were collected for broodstock from 1997 to 2002 as they passed the Roza Dam Adult Monitoring Facility (Roza Dam, Fig. 1). Adults were spawned at CESRF, and eggs and fry were reared at the facility for approximately 16 months. Juveniles were then transferred to three acclimation sites, which were designed to limit returns to the hatchery facility, expand the spatial influence of supplementation efforts, and permit related research on homing (Fig. 1). Following an acclimation period of two months, fish were allowed to volitionally begin their migration to the ocean. Approximately 80–90% of upper Yakima River Chinook spend 2 years in the ocean and return to freshwater at age 4 to reproduce (Knudsen et al. 2006).

First-generation hatchery fish began returning as adults in large numbers in 2001 and were allowed to spawn naturally. In 2002, CESRF spawned both wild and returning hatchery-origin adults to create the segregated (SEG) and integrated (INT) hatchery lines (Fig. 2). The segregated line relied solely on returning hatchery-origin adults for broodstock, and no fish were allowed to spawn naturally. In contrast, broodstock for the integrated line comprised only natural-origin fish, and all returning adults were free to spawn in the river. Natural-origin fish are those from the upper Yakima River population; however, beginning in 2005, returning adults were no longer 100% ‘wild’ due to possible influence from naturally spawning hatchery fish (Fig. 2).

### Sample collection

Tissue samples for DNA (operculum or axillary process) were collected from all fish during spawning at CESRF by facility staff or the Washington Department of Fish and Wildlife and stored in 100% ethanol in Olympia, WA. We subsampled tissues (*n* = 681) from the 1998 wild founders (2nd founding year; *P*_1_ Founders) and hatchery brood years 2002 (*F*_1_ Wild and *F*_1_ Hatchery), 2006 (*F*_2_ INT and *F*_2_ SEG), and 2010 (*F*_3_ INT and *F*_3_ SEG; Fig. 2). As most individuals mature at age 4, these brood years represent four generations of adults. We considered each generation of each hatchery line as separate ‘populations’ for the purpose of this study.

### DNA sequencing and genotyping

DNA was extracted using DNeasy Blood & Tissue kits (Qiagen, Valencia, CA, USA) following the animal tissue protocol. Restriction site-associated (RAD) libraries (Baird et al. 2008) were prepared, with 24–36 individuals per lane, using the restriction enzyme *Sbf1* and sequenced using the Illumina HiSeq 2000 platform.

RAD sequences were processed with *Stacks* (v. 1.09, Catchen et al. 2013). Reads were demultiplexed and trimmed to 74 base pairs in length because sequencing errors increased after that length. Samples that showed signs of contamination were removed from the analysis. All reads were then aligned to a reference database for Chinook salmon comprising 48 528 nonduplicated RAD loci, including 7146 mapped loci (Brieuc et al. 2014), using the ‘best’ option in *Bowtie* (v. 0.12.8, Langmead et al. 2009) and allowing up to three nucleotide mismatches. If a read aligned to multiple loci in the baseline, then those loci were omitted from downstream analyses. Following the *Bowtie* alignment, monomorphic and polymorphic loci were identified in *Stacks* using the bounded-error SNP calling model with default error rates and a minimum stack depth of 10 reads. Loci were then filtered for those with two alleles. In an effort to correct for bias in genotype calls due to differences in read depth between two alleles at a locus, all bi-allelic loci were re-genotyped for each individual with a custom Python script. This script designates a genotype as heterozygous if both alleles had a minimum depth of two and a combined depth >10 reads (Brieuc et al. 2014). Next, loci were filtered and retained if they had a minor allele frequency ≥0.05 in at least one population. These criteria were used to reduce error associated with genotyping duplicated loci that have been retained in the salmonid genome following a whole duplication event, and to provide stringency for the detection of signatures of selection. Individual samples were removed if they had ≥50% missing genotypes across all filtered loci.

### Heterozygosity and population differentiation

Observed (H_o_) and expected (H_e_) heterozygosity for each population, and tests for Hardy–Weinberg equilibrium (HWE) at each locus, were computed in the R-package *adegenet* (v. 1.3-9; Jombart 2008). Tests for HWE were conducted using the Monte Carlo procedure and 1 × 10^5^ permutations. The expected false discovery rate (*q*-value) for each locus was computed to correct for multiple testing using the R-package *qvalue* (v. 1.28.0; Storey 2002). A locus was deemed significantly out of HWE when the *q*-value was <0.05. Loci out of HWE, however, were retained for analyses because they may be of interest to present and future studies.

Two approaches were used to measure genetic change between each of the lines across generations. First, genetic differentiation, *F*_*ST*_, was calculated between populations and at each locus using Weir and Cockerham’s unbiased estimator in *Genepop* (v. 4.1, Weir and Cockerham 1984; Raymond and Rousset 1995). Significance of pairwise population *F*_*ST*_ comparisons was determined from tests of genotypic differentiation performed in *Genepop* using default parameters.

Second, a discriminant analysis of principal components (DAPC) was used to visualize temporal changes in genetic relationships between the lines and to identify loci that contribute most to population separation. Principal components analysis (PCA) is not optimal for the analysis of population structure because it maximizes total genetic variation but does not account for underlying genetic structure (Jombart et al. 2010). On the other hand, discriminant analysis (DA) is affected by correlations between variables (loci) and requires the number of variables to be less than the number of objects (individuals) (Borcard et al. 2011). These constraints are problematic for analysis of genomic data because some loci are physically linked, and the number of loci is typically much greater than the number of individuals. DAPC overcomes these drawbacks by combining PCA and DA. First, PCA is used to identify synthetic, uncorrelated variables (principal components or PCs) that maximize the amount of genetic variation explained in the data. PCs are then retained as variables for DA; the actual number of PCs retained can be chosen to ensure that the number of variables is less than the number of objects. DA is subsequently performed on the retained PCs to explore divergence between groups.

DAPC was conducted in the R-package *adegenet* using all individuals and loci. As the analysis requires a complete data set, missing values were replaced by the mean frequency of the corresponding allele, computed on the whole set of individuals. To avoid over-fitting the discriminant functions, the *optim.a.score* function was used to identify the optimal number of principal components to retain in the first step of the analysis based on the difference between observed and random discrimination.

Loci that contribute most to group separation, which we refer to as discriminatory loci, were identified using the *snpzip* function. The contributions of loci to the DAPC, or loadings, were first used to compute a distance matrix between loci. A hierarchical clustering analysis using the median clustering method was then performed on the distance matrix to separate loci into those that contribute to group divergence and those that do not. Discriminatory loci were compared to explicit tests for molecular signatures of selection to provide support for loci consistent with adaptive divergence.

### Effective number of breeders

Effective number of breeders, *N*_*b*_, was compared between hatchery lines as a proxy metric for estimating the effects of genetic drift in each population. *N*_*E*_ *Estimator* (v. 2.01, Do et al. 2014) was used to obtain estimates of *N*_*b*_ by the linkage disequilibrium (LD) and temporal methods. To reduce potential bias due to selection, loci that were identified as outliers by *F*_*TEMP*_ and *Bayescan*, as well as DAPC discriminatory loci, were omitted. For each year sampled, we used only four-year-old adults, which represented a single cohort of individuals and thus provided the most appropriate measure of *N*_*b*_.

For the LD estimates, we assumed random mating and used an allele frequency restriction of 0.05. Bias due to physical linkage was removed by excluding calculations between pairs of loci on the same chromosome (Larson et al. 2014). Estimates of *N*_*b*_ and 95% parametric confidence intervals were subsequently calculated for all populations (Larson et al. 2014). However, estimates of *N*_*b*_ obtained by the LD method may be biased by overlapping generations and fluctuating population size (Waples et al. 2014). We accordingly adjusted our estimates of *N*_*b*_, as the study population was subject to both sources of potential bias (Fig. 3), based on principles in Waples et al. (2014). Briefly, annual census data were first grouped into generations comprising four years each, as 80–90% of this population matures at age four (Knudsen et al. 2006). For each generation, we calculated the harmonic mean of total number of spawners per year (*N*_census_), as low return years within a generation have a disproportionate effect on *N*_*b*_, and multiplied the mean by four to obtain total number of spawners per generation (*N*_gen_). We then calculated the weighted harmonic mean of total spawners for the previous three generations (Wt. *N*_gen_), as past demographic history influences LD estimates of *N*_*b*_ (Waples et al. 2014). Previous generations, starting from the most recent, received weights of 1/2, 1/4, and 1/8 because half of existing LD decays each generation (Waples et al. 2014). We then divided Wt. *N*_gen_ by *N*_census_ for the year of interest. This ratio approximates the ratio of *N*_*e*_/*N*_*b*_, assuming *N*_e_/*N*_gen_ is proportional to *N*_*b*_/*N*_census_. As *N*_*b*_ estimates are a function of the harmonic mean of N_e_ and true *N*_*b*_ (Waples et al. 2014), this ratio was then used to calculate bias and adjust our *N*_*b*_ estimates. Full calculations are shown in Table S6.

For the temporal method, *N*_*b*_ and 95% confidence intervals were calculated using the *P*_1_ founders and three hatchery generations based on all non-outlier loci. We followed the Jorde and Ryman method, Plan II sampling, and used an allele frequency restriction of 0.05.

### Detection of outlier loci consistent with adaptive divergence

Given that *F*_*ST*_ outlier tests are known to exhibit a high rate of false-positive results (Lotterhos and Whitlock 2014), three independent tests for genomic regions indicative of diversifying selection were conducted to explore the potential role of selection in divergence of the two lines from the founder population. In our final interpretation, emphasis was placed on outlier regions that were identified by multiple tests, and were significantly divergent across multiple generations.

#### *F*_*TEMP*_ method

*F*_*TEMP*_ (Therkildsen et al. 2013) is designed to detect selection in a single population sampled across multiple generations by simulating genetic drift over time. The method is a modification of the widely used *Fdist* approach (Beaumont and Nichols 1996), and is based on an island model with drift but no migration. We parameterized simulations of genetic drift using estimates of *N*_*b*_ derived from the study populations, and explicitly identified loci that exceeded neutral expectations (i.e., consistent with signatures of adaptive divergence) in each hatchery line. Drift was simulated at 1 × 10^6^ loci for four generations. The model was first run using *N*_*b*_ estimates obtained by the temporal method using all loci (*N*_*b*_ values of 459 and 61 for the integrated and segregated hatchery lines, respectively). Yet, *N*_*b*_ estimates can themselves be biased by loci under selection. Therefore, *F*_*TEMP*_ outlier loci identified in the first run, as well as *Bayescan* outlier and DAPC discriminatory loci, were removed, and second estimates of *N*_*b*_ were calculated using only non-outlier loci. Estimates of *N*_*b*_ using nonoutlier loci were 466 and 69 for the integrated and segregated hatchery lines, respectively, and final *F*_*TEMP*_ simulations incorporated these *N*_*b*_ estimates. However, to test the sensitivity of our results to *N*_*b*_, we also ran *F*_*TEMP*_ simulations using the following estimates: (i) *N*_*b*_ estimates obtained from individuals of all ages (*N*_*b*_ of 388 and 59 for INT and SEG lines, respectively) instead of those calculated using only 4-year-olds; (ii) estimates of N_e_ instead of *N*_*b*_ (approx. 4*N_*b*_, Waples 2002), and (iii) *N*_*b*_ estimates for the opposite hatchery line (i.e., *N*_*b*_ of 466 for the SEG line and 69 for the INT line).

Sample sizes (*n*) per generation were also parameterized in the *F*_*TEMP*_ simulations, where sample sizes equaled the harmonic means of the number of individuals sequenced in each generation (61 and 57 for the integrated and segregated lines), but randomly varied between 0.8*n and *n* to account for variation in the number of individuals genotyped per locus per generation. One thousand final *F*_*TEMP*_ simulations were performed to account for variability between runs. To correct for multiple testing and control the false discovery rate (Storey 2002), the q-value of each locus was computed from its corresponding *P*-value using the R-package *qvalue*. A locus was considered significant if its *q*-value was <0.05 in at least 95% of the final simulations. Significant loci showed greater temporal allelic variation than is expected under genetic drift alone.

#### Bayescan

We also employed a Bayesian approach to identify outlier loci using the program *Bayescan* (v. 2.1; Foll and Gaggiotti 2008). However, this approach assumes that the sampled populations evolved independently from a common ancestral population; thus, it is not ideally suited for temporal data. In addition, the power of *Bayescan* to detect outlier loci is reduced with few populations (<6), low sample sizes (<30), and low neutral *F*_*ST*_ values (<0.01) (Foll and Gaggiotti 2008). Despite these limitations, we used *Bayescan* because it has lower type I and type II errors than other available methods (De Mita et al. 2013). The program was run with all populations combined with default parameters. A locus was considered to be an outlier if its *q*-value was <0.05 in each of five independent runs.

#### Test for outlier regions of the genome: sliding window analysis

Moving averages of pairwise *F*_*ST*_ values between each hatchery line and the *P*_1_ founders at mapped markers were calculated using a kernel smoothing sliding window approach (Hohenlohe et al. 2010; Brieuc et al. 2015). Each chromosome was divided into 100 windows spanning 18 cM each; these parameters were optimized to detect small regions of divergence but minimize background noise due to sampling variance. Contributions of individual loci to the *F*_*ST*_ average were weighted by their distance from the center of the window. A null distribution with 95% confidence intervals was calculated for each window using 1 × 10^6^ replicates of randomly sampling *F*_*ST*_ values from all mapped loci, with replacement, where sample size was based on the number of loci within each window. The sliding window analysis was performed separately on the *F*_1_, *F*_2_, and *F*_3_ generations of each hatchery line. Regions of significantly elevated divergence were identified as those where the moving average exceeded the 95% confidence interval of the null distribution.

### Outlier alignment and gene function

All loci located within the genomic regions that exhibited overlap among the three tests of adaptive divergence (summarized in Table1) were aligned to the rainbow trout genome (Berthelot et al. 2014) using *Bowtie* (Langmead et al. 2009) to identify genes associated with divergence and potential targets of domestication selection. *BLAST2GO* (Conesa et al. 2005; Götz et al. 2008) was then used to conduct a *BLAST* (Altschul et al. 1990) search on the NCBI nr public database for the rainbow trout gene coding sequences that were identified. We used an *e*-value threshold of 1 × 10^−10^ in *BLAST* searches. Gene ontology (GO) terms, GO Slim terms, and functions associated with each gene were subsequently identified.

**Table 1:** Genomic regions that exhibited overlap among all three tests of adaptive divergence. The table indicates the number of generations (Gens) that a portion or all of each region was identified as a region of high divergence by the sliding window (SW) analyses for the integrated (INT) and segregated (SEG) lines, and the number of outlier loci within each region that were identified by *F*_*TEMP*_ or *Bayescan*

Region	Chromosome	Map position (cm)	Gens SW INT	Gens SW SEG	*F*_*TEMP*_INT	*F*_*TEMP*_SEG	Bayescan
1	Ots04	37.52–43.15		2		2	2
2	Ots05	87.43–95.05	1	3		1	1
3	Ots06	94.64–100.65	1	1		1	1
4	Ots11	57.77–71.46	2	3	1	2	1
5	Ots12	26.14–35.29	1	2		2	2
6	Ots15	132.33–139.26	1	1		1	1
7	Ots20	93.65–99.49	1	2		2	1

## Results

### DNA sequencing and genotyping

We identified 9410 bi-allelic RAD loci, including 4405 loci that aligned to the Chinook salmon linkage map and had a minor allele frequency >0.05 in at least one population (Table S1). A total of 413 individuals were genotyped at >50% of these loci and retained for analysis (Table S1). Sample sizes for the *P*_1_ founders and each generation of integrated and segregated hatchery lines are summarized in Table S2.

### Heterozygosity and population differentiation

Observed (*H*_o_) and expected (*H*_e_) heterozygosity were similar between the integrated and segregated hatchery lines within each generation, although a small decrease was observed over time (Table S2). All populations had <3% of loci that deviated from Hardy–Weinberg equilibrium. Loci that deviated from HWE were included in further analyses because they may be of interest to this study.

Population genetic differentiation, *F*_*ST*_, for each of the generations compared to the *P*_1_ founders was low in all pairwise comparisons, ranging from 0 to 0.0108 (Table S3). The *F*_1_ hatchery fish and segregated line steadily diverged from the *P*_1_ founders over time; the *F*_2_ and *F*_3_ SEG populations were significantly differentiated from the founders (*F*_*ST*_ = 0.0049 and 0.0108 respectively, *P* < 0.001). In contrast, the integrated line did not exhibit the same temporal trend of increasing genetic divergence. However, *F*_*ST*_ compared to the *P*_1_ founders was significant for the *F*_2_ and *F*_3_ INT populations (*F*_*ST *_= 0.0026 and 0.0022, respectively, *P* < 0.001). *F*_*ST*_ between the *F*_3_ SEG and *F*_3_ INT populations was also significant (*F*_*ST*_ = 0.0093, *P* < 0.001), indicating divergence in the two hatchery lines.

**Figure 4:**
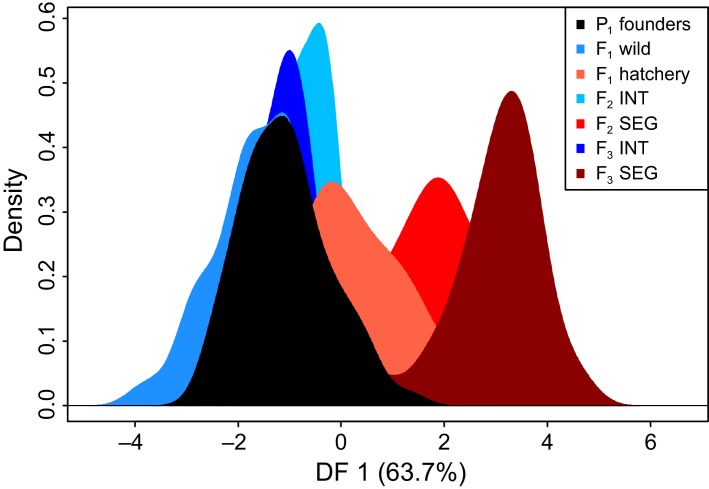
Density plot of individuals from the wild founders (*P*_1_ Founders, black) and three generations of the integrated (INT, blue colors) and segregated (SEG, red colors) hatchery lines along the first discriminant function from the discriminant analysis of principal components (DAPC).

The first step of the discriminant analysis of principal components (DAPC) identified 58 PCs to retain, which explained 29.3% of the observed genetic variation among individuals. A plot of the kernel density estimates of individuals along the first discriminant function, which explained 63.7% of the retained variation, showed separation of the *F*_1_ hatchery fish and segregated line from the *P*_1_ founders (Fig. 4). The segregated line became more divergent over time; in comparison, the *F*_1_ wild group and integrated hatchery line clustered closely with the *P*_1_ founders. The second discriminant function explained 13.6% of the retained variation and, to a minor extent, represented temporal variability within the two lines. We identified 25 loci that contributed most to separation along the first discriminant function based on hierarchical clustering analysis (termed discriminatory loci; Table S8). A majority of the discriminatory loci (17 of 25) did not overlap with outlier loci identified by explicit tests of selection and thus were inferred to be selectively neutral.

### Estimates of effective number of breeders

**Figure 5:**
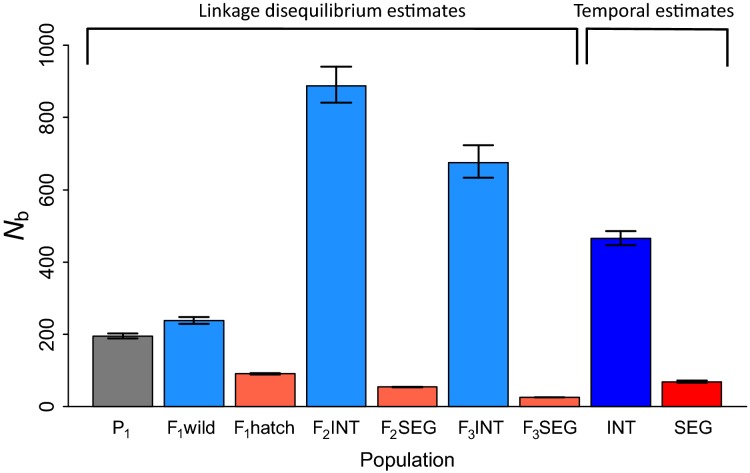
Effective number of breeders, *N*_*b*_, and 95% confidence intervals estimated by the linkage disequilibrium (LD) and temporal methods for the *P*_1_ Founders (gray) and integrated (blue) and segregated (red) hatchery lines. The LD method allows estimation of *N*_*b*_ for every generation, while the temporal method yields a single estimate for the entire sampling period. LD estimates are adjusted for physical linkage and potential bias caused by overlapping generations and fluctuating population size.

Estimates of the effective number of breeders, *N*_*b*_, obtained by the LD method for each sample year largely reflected the effective number of parents that produced the samples (Waples 2005), but also had contributions from previous generations (Waples et al. 2014). Bias-adjusted estimates of *N*_*b*_ from the natural population revealed a 3–4 fold increase following establishment of the integrated hatchery line (Fig. 5; Table S5a), although this period also experienced higher adult numbers returning to the basin (Fig. 3). Estimates of *N*_*b*_ in the segregated line declined steadily over time and, in the latter two generations, were over an order of magnitude lower than in the integrated line (Fig. 5; Table S5a), suggesting that genetic drift may have driven the observed genetic divergence. Differences between the two lines were not unexpected, as the average broodstock sizes were 363 (SD = 56) and 85 (SD = 15) for the integrated and segregated lines, respectively. Interestingly, wild fish that were spawned in the hatchery in 1998 had a lower *N*_*b*_ estimate than the wild fish that reproduced in the river, despite having similar census sizes and the same population history (Table S5a).

The temporal method provided single estimates of *N*_*b*_ for the integrated and segregated lines across the sampled generations (Fig. 5; Table S5b). Temporal estimates closely agreed with the harmonic means of the LD estimates and showed that the integrated line had a larger *N*_*b*_.

### Detection of outlier loci and chromosomal regions of high divergence

*F*_*TEMP*_ identified 228 temporal outlier loci in the segregated line and 80 in the integrated line (Table S8). Thirty-two loci were identified as outliers in both lines. *F*_*TEMP*_ results did not change considerably when run with the *N*_*b*_ estimates obtained from individuals of all ages. Running *F*_*TEMP*_ with estimates of *N*_e_ instead of *N*_*b*_ did not alter results for the integrated line, but it significantly increased the number of temporal outliers in the segregated line. In addition, there were 369 *F*_*TEMP*_ outliers in the segregated line but just 48 outliers in the integrated line when the *N*_*b*_ estimates for one hatchery line were used for the opposite line. These results indicated that the numbers of outlier loci detected in the segregated line were consistently higher than in the integrated line across a range of *N*_*b*_ values, and that using the single cohort *N*_*b*_ estimates instead of *N*_*e*_ was a conservative choice.

The second analysis, conducted using *Bayescan*, identified 75 outlier loci across all combined populations (Table S8). As the *Bayescan* analysis required combining populations, outlier loci could not be attributed to a specific hatchery line. However, there was considerable overlap with *F*_*TEMP*_ results. Twenty-eight *Bayescan* outlier loci were also *F*_*TEMP*_ outliers in both hatchery lines, while an additional 17 and 22 *Bayescan* loci were *F*_*TEMP*_ outliers in only the integrated or segregated lines, respectively.

**Figure 6:**
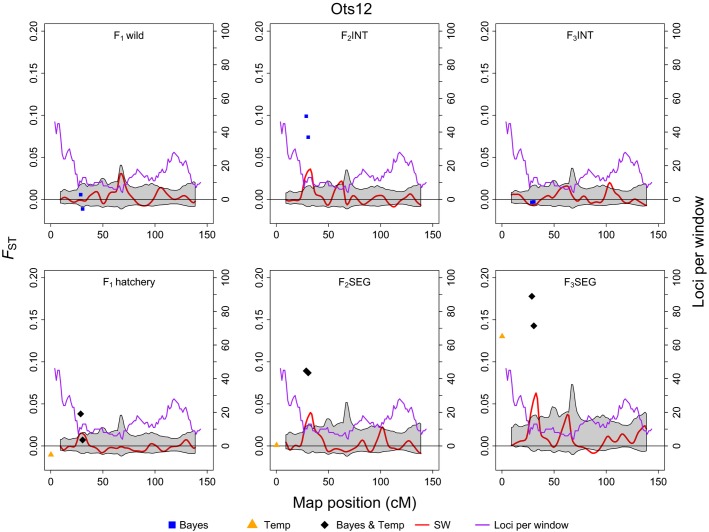
Loci and regions of the genome showing signatures of adaptive divergence, based on pairwise *F*_*ST*_ compared to the *P*_1_ founders, on chromosome Ots12 for the integrated (top panel) and segregated (bottom panel) hatchery lines through the *F*_1_, *F*_2_, and *F*_3_ generations. Blue squares are loci that were identified as outliers with *Bayescan*, orange triangles are outliers identified by *F*_*TEMP*_, and black diamonds are loci identified by both *Bayescan* and *F*_*TEMP*_. The red line represents the kernel smoothed moving average of *F*_*ST*_ and the gray-shaded area is the 95% confidence interval. The purple line shows the number of loci within each sliding window of the moving average.

Lastly, sliding window analyses conducted on the 4405 mapped loci identified several regions of significantly elevated *F*_*ST*_ values in both hatchery lines (Table S9). However, regions of high divergence in the segregated line were more temporally stable than those in the integrated line; that is, more regions were consistently identified across the *F*_1_, *F*_2_, and *F*_3_ generations. Five genomic regions had significantly elevated *F*_*ST*_ values in two of the three generations within the segregated line, and four regions were significantly high in every generation. In contrast, the integrated line contained three regions that were significantly elevated in two generations, and no region was divergent across all generations. Furthermore, plots of per locus *F*_*ST*_ values for markers positioned along the chromosome revealed that divergence from the *P*_1_ founders increased over time at some regions in the segregated line, and these regions also contained loci that were identified as outliers by other tests (Fig. 6). This directional, temporal trend was not observed in the integrated line, although a region on Ots11 was significantly divergent in both hatchery lines in the *F*_1_ and *F*_3_ generations.

Overall, seven genomic regions exhibited overlap among all three tests of diversifying selection in the segregated line (Table1). One region showed consensus across tests in the integrated line, but this was also shared by the segregated line. These results, in conjunction with the temporal increase in *F*_*ST*_ at some regions, suggest that selection might also have contributed to divergence in the segregated line.

### Outlier alignment and gene function

Forty-three mapped loci were located within the seven genomic regions that exhibited overlap among the three tests of adaptive divergence, and 25 of these loci aligned to the rainbow trout (RBT) genome. Twenty-two loci, including five outliers, were located within or near annotated RBT genes. While the RBT genes had an array of functions, commonalities among genes were observed. Three genes, including one associated with an outlier locus, were related to the protein ubiquitin, which is important for protein degradation and immune response. Two other genes were also related to the function and response of the immune system. Three genes, with two linked to outlier loci, were involved in the utilization, breakdown, and reception of lipids, while another two genes, including one near an outlier locus, were essential for the production of ribose, a sugar that can be used for energy or the synthesis of nucleotides. Three genes were linked to neurotransmission, including one gene that coded for receptors of gamma-aminobutyric acid (GABA), a major inhibitory transmitter in the central nervous system, and a second gene that has been shown to induce clustering of GABA receptors. Lastly, two of the RBT genes, including one from an outlier locus, were linked to the development of photoreceptors and eye pigmentation. The shared functions of many genes provided insight into the potential targets of domestication selection. Alignment results, as well as gene functions with references, are summarized in Table S10.

## Discussion

The aim of this study was to evaluate the effectiveness of managed gene flow to reduce genetic divergence in supportive breeding programs. This goal was achieved by comparing genome-wide diversity in three generations of integrated and segregated Chinook salmon hatchery lines to their founding wild population. Genetic distance measures at 9410 loci showed that divergence in the segregated line was significant by the second generation. Much of this change can be ascribed to genetic drift, as suggested by the small numbers of effective breeders. However, we also found evidence for a temporal trend in divergence at specific genomic regions, consistent with domestication selection. In contrast, genetic divergence in the corresponding integrated line was marginal over three generations, suggesting that the use of natural-origin broodstock was effective at reducing genetic change in the short term. By comparing contrasting regimes, the results illustrate the range of possible outcomes that may occur when using deliberate gene flow to mitigate genetic impacts in captive breeding programs. This information is therefore highly relevant when weighing the relative risks of different captive rearing strategies.

Like many such studies on supportive breeding programs (Araki et al. 2007; Hess et al. 2012; Anderson et al. 2013; Christie et al. 2014), this study does not have a contemporary control population to which the hatchery lines can be compared. After the first hatchery generation, the upper Yakima population was no longer ‘wild’ due to influence from the integrated line. Therefore, ongoing evolutionary processes in the natural environment cannot be readily discriminated from those in the hatchery. However, the comparisons to the wild founding population strongly suggest that divergence in the segregated line was primarily due to repeated exposure to the hatchery environment, as differentiation did not occur in the integrated line.

It is also important to note that the contribution of integrated hatchery fish to natural population productivity has not been fully quantified. Higher adult returns in the natural population were observed following initiation of the hatchery (Fig. 3), but this increase might be attributed to supplementation, a favorable shift in environmental conditions (Mantua et al. 1997; Fast et al. 2015), or a combination of factors (Scheuerell et al. 2015). Previous studies suggest that hatchery-reared individuals tend to be less fit than naturally-born fish (Araki et al. 2007; Milot et al. 2013; Christie et al. 2014), but successfully spawning hatchery fish can still add to natural production. In this system, there are three lines of evidence that suggest that hatchery fish contributed to population productivity. First, the proportion of hatchery fish spawning in the wild reached 70–75% in some years (Figs2 and 3) and, given these proportions, it is highly likely that these fish supported subsequent generations. Second, nest (redd) abundance and spatial distribution significantly increased following supplementation efforts, an increase that exceeded numbers observed in a nearby unsupplemented river (Fast et al. 2015). Third, comparisons of female reproductive traits (Knudsen et al. 2008) and breeding success experiments within an artificial stream (Schroder et al. 2008, 2010) revealed little difference between wild and first-generation hatchery salmon, although offspring of wild females had significantly higher survival to the fry stage (5.6%, *P* = 0.04; Schroder et al. 2008). A full understanding of the contribution of hatchery-reared fish to population productivity will be gained through complementary pedigree-based studies.

An ideal experiment would have compared lines with equal numbers of broodstock. For example, in theoretical treatments on the effects of different management strategies on genetic change in supportive breeding programs, Duchesne and Bernatchez (2002) found that increasing the census size of the captive population had the most influence on reducing the inbreeding coefficient compared to other tactics such as changing the proportion of wild individuals in the broodstock or modifying the numbers of captive fish released. Here, the numbers of adults spawned in the segregated line were typically a quarter of those in the integrated line, an outcome of the fact that the Cle Elum facility serves broader restoration goals beyond the experiment. This difference in broodstock size partly explains the divergence from the founder population observed in the segregated line, and it is likely that simply increasing the broodstock size would have reduced divergence. However, one of the major objectives of managed gene flow is to minimize genetic drift. This goal is partly achieved through taking advantage of both wild and hatchery production, effectively increasing the ‘rearing space’ of supportive breeding programs. Therefore, the effective size of integrated programs can be expected to exceed comparable segregated programs in cases where the hatchery or the wild component does not become a ‘sink’ for the ‘source’ population.

**Figure 7:**
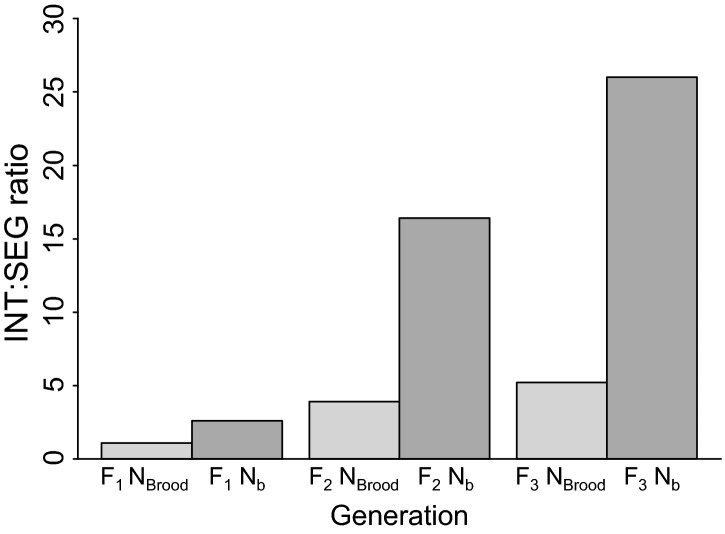
The integrated:segregated ratios of broodstock sizes (*N*_brood_ INT/*N*_brood_ SEG) and effective number of breeders (*N*_*b*_ INT/*N*_*b*_ SEG) for the *F*_1_, *F*_2_, and *F*_3_ hatchery generations. The *N*_b_ ratios were consistently higher than the *N*_brood_ ratios, indicating that differences in *N*_*b*_ between the hatchery lines were primarily due to managed gene flow and not differences in broodstock sizes.

Evaluations of the broodstock census size and effective number of breeders across the integrated and segregated lines provide insight into the relative importance of gene flow in mitigating genetic divergence from the founder population. The ratio of broodstock census sizes (*N*_brood_ INT/*N*_brood_ SEG) for the two lines can be compared to the ratio of *N*_*b*_ estimates (*N*_*b*_ INT/*N*_b_ SEG) in each generation (Table S7). These ratios would be approximately equal if the differences in *N*_*b*_ estimates solely reflected differences in broodstock size. However, the ratios of *N*_*b*_ INT/*N*_*b*_ SEG were larger than the ratios of *N*_brood_ INT/*N*_brood_ SEG, particularly in the *F*_2_ and *F*_3_ generations (Table S7, Fig. 7). This simple comparison suggests that managed gene flow disproportionally increased *N*_*b*_ in the integrated line and reduced genetic divergence by incorporating the naturally spawning segment of the population.

There is an interesting aspect of this study that illustrates the temporal variation in the effects of hatchery rearing on *N*_*b*_. The 2002 (*F*_1_ period) estimates reflect the *N*_*b*_ of fish that spawned in 1998 (*P*_1_ period). In the P_1_ period, approximately equal numbers of wild fish spawned in the river and in the hatchery, yet the *N*_*b*_ of fish in the river was over two times greater than the *N*_*b*_ of fish in the hatchery. The result suggests that in this generation, the hatchery environment caused higher variance in reproductive success and decreased *N*_*b*_, which is consistent with other studies of effective size in hatcheries (reviewed in Naish et al. 2008; Christie et al. 2012; Naish et al. 2013). In contrast, the ratio *N*_*b*_/*N* decreased in the *F*_2_ and *F*_3_ generations of the integrated line despite higher *N*_*b*_ estimates relative to the segregated line, suggesting that ecological influences such as higher competition on the spawning grounds (Fleming and Gross 1994) or limited carrying capacity might have influenced this ratio as the population increased in size. Overall, these results emphasize the dynamic impacts of both natural processes and hatchery rearing on effective size, the significance of multigenerational monitoring, and the need to understand the factors influencing the success of supportive breeding programs as a whole.

Multiple lines of evidence and consistent temporal trends in the segregated line provided compelling support for some genomic regions involved in domestication. In contrast, outlier regions detected in the integrated line had minimal overlap among tests and lacked temporal trends. Tests for *F*_*ST*_ outliers are known to exhibit high false-positive rates (Lotterhos and Whitlock 2014), but they continue to be used because they serve as useful starting points for further investigations (Storz 2005). Here, we reduced the likelihood of false-positive results using three independent tests for outliers (e.g., de Villemereuil et al. 2014), including one specifically designed to detect temporal divergence. Signatures of domestication selection are also difficult to detect, particularly in early generations (Lotterhos and Whitlock 2014; Mäkinen et al. 2015), and power is reduced with few populations in the analyses (Karlsson and Moen 2010). However, the results presented here are comparative and point toward greater and more rapid changes in the segregated line. More outliers were observed in the line with a smaller effective size, an interesting finding considering that selection is expected to be more efficient in larger populations (Wright 1931). Thus, the strength of selection must be greater in the hatchery than in the natural environment. This result has been observed in other small, fragmented populations (Koskinen et al. 2002). Finally, we note one outlier region that was divergent in the *F*_1_ and *F*_3_ generations of both hatchery lines. As this region was an outlier in the *F*_1_ wild group, the results might indicate a false positive or the action of natural selection. However, the region was also an outlier in the *F*_1_ hatchery and *F*_3_ groups, possibly providing evidence of selection in the hatchery at early life stages that has affected both lines. We cannot rule out additional regions of undetected selection in both lines, as it is currently not possible to survey the whole genome in Chinook salmon. Despite these potential limitations, outlier regions identified in the segregated line are supported by multiple lines of evidence and provide valuable insight for the genetic basis of adaptation to captivity.

Alignment of loci within outlier regions to the rainbow trout genome and classification of gene functions identified possible targets of domestication selection. Multiple genes were related to the regulation and response of the immune system, which may be affected by the higher rearing densities in hatcheries. Many gene products, including those associated with outlier loci (ubiquitin, alpha–beta hydrolase, and ribokinase), were connected to the processing of proteins, lipids, and sugars and may be affected by hatchery feeding regimes. For example, in rainbow trout, the ubiquitin–proteasome pathway, which facilitates protein degradation, was affected by starvation (Martin et al. 2002) and feeding status (Seiliez et al. 2008). Additionally, differential gene expression was observed between groups of rainbow trout that were fed different diets (Panserat et al. 2009). Two other gene products were important for the development of photoreceptors and eye pigmentation (salmon are visual feeders). Together, these functions suggest that domestication selection may be occurring in the hatchery environment due to differences in food availability and composition.

The two GABA-related genes, one that coded for GABA receptors and a second that induces clustering of GABA receptors, suggest another potential target of domestication selection. GABA has been shown to stimulate the secretion of two gonadotropins, GTH-1 and GTH-2, in rainbow trout (Mañanos et al. 1999), and these gonadotropins have been shown to stimulate gonad growth in juveniles (Suzuki et al. 1988). Domestication selection acting on this pathway may explain changes in rates of early male maturation commonly observed in hatchery populations of Chinook salmon (Larsen et al. 2010; Harstad et al. 2014). Furthermore, early male maturation is positively correlated with cumulative growth (size at release, Harstad et al. 2014). Domestication selection likely targets polygenic traits, and here, there is evidence that such selection may affect both the GABA-related genes and the genes that process proteins, lipids, and sugars.

The results reported here add to the growing number of studies that have, to date, typically focused on the relative performance of one or two generations of captive individuals released into the wild, and only under a single broodstock management regime. In salmon species, a few such studies documented minimal differences between hatchery and wild fish, including comparable reproductive success (Anderson et al. 2013), breeding success in an artificial channel (Schroder et al. 2008, 2010), reproductive traits (Knudsen et al. 2008), and spawning distributions (Dittman et al. 2010). However, many studies have found significant differences in hatchery fish, including reduced response or increased vulnerability to predation (Fritts et al. 2007), lower survival (McGinnity et al. 2003), differences in growth rate and morphology (McGinnity et al. 2003; Busack et al. 2007), and reduced reproductive success (Araki et al. 2007, 2009; Christie et al. 2014). Yet, the genetic basis of these differences, and the rate at which they occur, remain unclear. Using an experimental system that included contrasting lines founded from a single population and sampled extensively over multiple generations, we began to address these uncertainties by estimating the rate of genetic change in hatchery fish with unprecedented resolution. While the magnitude of divergence observed in the segregated line was small, it is important to consider that this change occurred after only three generations of captive rearing. If the trend of divergence continues, then *F*_*ST*_ could equal or exceed values typically observed between natural Chinook salmon populations (e.g., Brieuc et al. 2015) within another three to five generations. Thus, we consider the rate at which genetic differentiation occurred to be noteworthy and of significant interest to conservation programs. We also identified processes driving change in the segregated line, including the first possible indicators consistent with domestication selection observed in Pacific salmon hatchery populations.

Determining the impact of different captive rearing approaches on population productivity in species of conservation interest is central to measuring their success, but empirical studies on such species are usually constrained by the natural systems in which they operate. An ideal experiment would demonstrate that integrated fish have greater fitness than segregated fish in the natural environment. However, permitting spawning of the segregated fish in the same environment as the integrated fish would promote gene flow between the lines, and negate both the experiment and the concurrent restoration efforts in the Chinook salmon system we have studied. Therefore, it was necessary to use proxies for fitness by identifying signatures of selection and measuring effective population size, consistent with best practices in genetic monitoring (Schwartz et al. 2007). These measures can be further enhanced by integrating genome-wide association studies with phenotypic measures of fitness.

This study is the first to empirically compare multigenerational consequences of alternative management approaches in a nonmodel species of conservation interest using genome-wide surveys, and adds to the range of available tools for assessing risks associated with supportive breeding. Multigenerational observations are advantageous because processes occurring in the wild, including natural selection, could mitigate or exacerbate the effects of captive rearing over time. Our findings provide the first empirical demonstration that using natural-born parents in captive breeding programs is more effective at reducing genetic divergence than using only captive-born individuals. Interpreting these findings in a management context can be challenging on one hand, because determining ‘acceptable’ levels of divergence depends in part on a clear link between the molecular measures we report and population productivity. On the other hand, the study is comparative in nature and considers a range of possible outcomes, an approach that is highly relevant to risk assessment in realistic scenarios (Waples 1999; Duchesne and Bernatchez 2002; Ford 2002; Waples and Drake 2004; Naish et al. 2008). The experimental system we evaluated therefore provides guidance on ‘best practices’ in supportive breeding. Conservation plans should thus consider establishing captive breeding programs using wild broodstock before the source population becomes too small, so that the benefits of starting such a program outweigh the demographic and genetic costs of removing individuals from the natural environment.

## Data archiving statement

Data available from the Dryad Digital Repository: http://dx.doi.org/10.5061/dryad.v33f0.

## Supporting Information

**Table S1.** Lists of the 9410 RAD loci and 413 individuals, with their populations, analyzed in this study. **Table S2.** Description of wild founders and each generation of the integrated (INT) and segregated (SEG) hatchery lines, including year sampled, the number of adults used as broodstock, the number of broodstock sampled in this study, the final sample size retained for each population after removing individuals with >50% missing genotypes, observed (*H*_o_) and expected (*H*_e_) heterozyosity, and the number of loci that deviated from Hardy-Weinberg equilibrium after correcting for multiple tests. **Table S3.** Pairwise population differentiation (*F*_*ST*_) compared to the *P*_1_ Founders and between the two lines within each generation. **Table S4.** *F*_*ST*_ per locus for each population when compared to the *P*_1_ Founders. **Table S5.** (a) Spawning groups and census sizes (*N*_census_) to which estimates of effective number of breeders, *N*_*b*_, apply, the proportions of *N*_census_ that were of hatchery origin (*P*_hatchery_), and the years and populations from which *N*_*b*_ estimates were obtained using the linkage disequilibrium (LD) method. *N*_*b*___LD_ (95% CI) are the LD estimates with 95% confidence intervals, Bias is the approximate bias in the LD estimates resulting from overlapping generations and fluctuating population sizes, *N*_*b*___Adj_ (95% CI) are the bias-adjusted estimates with 95% confidence intervals, and *N*_*b*___Adj_/*N*_census_ is the estimated ratio of successful spawners to potential spawners. (b) Effective number of breeders (95% CI) for both hatchery lines from 1998–2010 estimated using the temporal method. **Table S6.** Bias calculations for *N*_*b*_ estimates generated by the LD method in *NeEstimator* for the (a) river (wild and integrated line) and (b) hatchery (segregated line). **Table S7.** Estimated contribution of naturally spawning fish (i.e. managed gene flow) to *N*_*b*_ in the integrated line. **Table S8.** Discriminatory loci identified for DF1 of the DAPC, and outlier loci identified by *Bayescan* and *F*_*TEMP*_. **Table S9.** Regions identified by the sliding window analyses as showing significantly elevated divergence from the *P*_1_ founders. **Table S10.** (a) Alignments to the rainbow trout (RBT) genome, *BLAST* results, gene functions, and gene ontologies (GO Slim Terms) for all mapped loci within the seven genomic regions that exhibited overlap among the three tests of adaptive divergence (Region of Overlap, described in Table1). (b) Complete references, listed in alphabetical order, for the gene functions described in Table S10a.
